# Infectious origins of childhood leukemia

**DOI:** 10.18632/oncotarget.4630

**Published:** 2015-06-24

**Authors:** Srividya Swaminathan, Markus Müschen

**Affiliations:** Department of Laboratory Medicine, University of California San Francisco, CA, USA

**Keywords:** B cell development, acute lymphoblastic leuke-mia, inflammation

Pediatric pre-B acute lymphoblastic leukemia (ALL) may develop from prenatal chromosomal translocations acquired *in utero*. For instance, the *ETV6-RUNX1* gene rearrangement that occurs in ~25% of childhood ALL clones is found in the umbilical cord blood and Guthrie blood spots of 1 in 100 healthy newborns [1]. Despite a high frequency of occurrence of *ETV6-RUNX1* in healthy neonates, only 1 in 14,000 carriers develop overt leukemia [2]. Other examples of pre-natally acquired lesions that are also found in healthy newborns include *MLL*- and *BCR-ABL1* gene rearrangements [1]. Studies on identical twins carrying pre-natal genetic lesions revealed significant differences in latency of leukemia development [1, 3]. These observations corroborated that a prolonged clonal evolution process precedes overt leukemogenesis in *ETV6-RUNX1* carriers [3]. However, until recently, the molecular mechanisms accelerating this clonal evolution remained unclear.

Clonal evolution processes enable pre-leukemic pre-B cells to acquire secondary mutations that in some cases confer a competitive survival advantage over other pre-B cell clones. Pre-leukemic clones can, therefore accumulate in the bone marrow and acquire additional lesions that will ultimately give rise to overt leukemia. A recent study showed the clustering of mutation hot spots in B cells to regions frequently targeted by the enzymes Activation Induced Cytidine Deaminase (AID) and Recombination Activation Genes 1 and 2 (RAG1-RAG2) [4]. Interestingly, AID and RAG1-RAG2 are genetic modifiers of the immunoglobulin (Ig) genes that are naturally expressed during B-lymphopoiesis. Although AID and RAG1/RAG2 are thought to be segregated to early (RAG1/RAG2) and late (AID) stages of B cell development, respectively, others and we recently showed that the two enzymes could be concurrently expressed during early B-lymphopoiesis. Our experiments identified the early B cell subset that is particularly vulnerable to such concomitant expression of AID and RAG1-RAG2. Further investigations revealed that a decrease in the B cell responsiveness of this pre-B subset to the cytokine IL-7 effected the combined activation of both the enzymes. We showed that human B cells from children lacking a functional IL-7 receptor (IL-7R) displayed both increased expression and activity of AID. These results demonstrated that AID activation in both mouse and human early B cell compartments follows a common molecular paradigm [5].

RAG1 and RAG2 induce DNA double-strand breaks during V(D)J recombination in pro- and pre-B cells and their role as drivers of pre-B leukemogenesis was recently elucidated [6]. Extending these findings, our analysis of pre-B ALL patients revealed replacement of the variable region (V^H^) of the Ig heavy chain predominantly in the *ETV6-RUNX1* group [5]. V_H_ replacement is a phenomenon that is induced by RAG1-RAG2-driven DNA recombination. Aside from this, we observed DNA mutations in Ig genes of pre-B ALL patient samples, indicative of concomitant AID activity. Although concurrent activation of AID and RAG1-RAG2 in patient samples implicated a correlation between the two enzymes in the pathogenesis of leukemia, this as such did not prove that the enzymes causally induce overt leukemogenesis. Therefore, we next evaluated the requirement of AID and RAG1-RAG2 in leukemogenic transformation, and identified a condition that leads to massive activation of these enzymes in a pre-leukemic B cell.

It is important to note that AID and RAG1-RAG2 expression increase dramatically during an infection, where both these enzymes diversify the antibody repertoire and improve its affinity to antigens from infectious pathogens. We therefore tested whether the pre-B cell subset that concurrently expresses AID and RAG1-RAG2 can respond to an inflammatory stimulus, such as LPS. We observed that pre-B cells require protection from IL7, which prevents aberrant activation of AID. In the absence of protective IL-7, these pre-B cells acquired responsiveness to LPS and strongly activated AID concurrently with RAG1-RAG2 enzymes.

**Figure 1:**
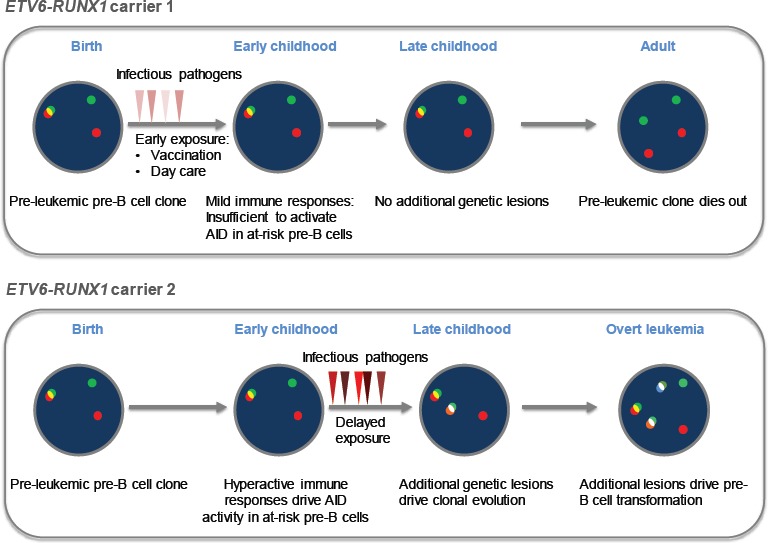
Mechanism of clonal evolution of ETV6-RUNX1 clones towards leukemia Mild infections in early childhood are insufficient to activate AID and thus do not allow clonal evolution of pre-leukemic *ETV6-RUNX1* clones. Chronic infections occurring for the first time in protected individuals during late childhood are severe as they induce AID activation. AID-induced genetic lesions in these protected individuals confer a survival advantage on the pre-leukemic *ETV6-RUNX1* clones giving rise to overt leukemia.

We developed IL7-dependent pre-B cell cultures as a disease model for *ETV6-RUNX1* pre-leukemia and tested the role of AID and RAG1 in the progression of pre-leukemic clones. To this end, we expanded *ETV6-RUNX1* pre-B cells from wildtype (AID and RAG1 expressing) mice, or from mice lacking AID (*Aid^−/−^Rag1^+/+^*) or RAG1 (*Aid^+/+^Rag1^−/−^*). We then challenged pre-B cell cultures by withdrawal of IL7 (loss of protection) and inflammatory stimuli (LPS) and transplanted pre-B cells that had undergone five cycles of –IL7/LPS challenge. Upon transplanting –IL7/LPS-treated *Aid^+/+^Rag1^+/+^* or *Aid^−/−^Rag1^+/+^* or *Aid^+/+^Rag1^−/−^* pre-B cells containing *ETV6-RUNX1* into NOD-SCID recipient mice, we observed that loss of either *Aid* or *Rag1* dramatically prolonged the latency and reduced the penetrance of leukemia in transplant recipients. This proved that AID and RAG1-RAG2 causally accelerate the clonal evolution of a pre-leukemic B cell towards overt leukemia. Our findings provide a molecular mechanism by which pre-leukemic clones carrying a prenatal genetic lesion such as *ETV6-RUNX1* can evolve through infectious and inflammatory stimuli ultimately leading to full blown leukemia [5, Figure 1].

The impact of inflammatory and infectious stimuli on leukemogenesis has been previously implicated in multiple epidemiological studies. These studies attributed the lack of exposure of infants to infections in the clean environments of developed societies to overactive B cell immune responses [7]. Moreover, multiple studies demonstrated that day-care attendance primed the immune system during early childhood. Such priming was hypothesized to prevent the exacerbation of B cell responses and the clonal evolution towards leukemia [Figure 1]. Although inflammation (LPS stimulation) seems to play a role in accelerating pre-B leukemogenesis in our model, further experiments mimicking actual infections must be conducted in mice. Moreover, it is crucial to test whether leukemogenesis is accelerated in individuals infected with restricted classes of pathogens, such as bacteria, viruses and parasites. A crucial factor to take into consideration is that not all classes of pathogens may uniquely activate AID in pre-B cells. Along these lines, it would be insightful to test whether other members of the APOBEC family (apart from AID) have a role to play in the inflammatory and infectious origins of pediatric pre-B leukemia.
